# Common fixed point theorems for $(T,g)_{F}$-contraction in *b*-metric-like spaces

**DOI:** 10.1186/s13660-018-1802-z

**Published:** 2018-08-28

**Authors:** Dakun Yu, Chunfang Chen, Hong Wang

**Affiliations:** 1 School of Communication and Information Engineering, Shanghai University, Shanghai, P.R. China; 2 School of Sciences, Nanchang University, Nanchang, P.R. China

**Keywords:** 47H10, 54H25, *b*-metric-like spaces, Common fixed point, $(T,g)_{F}$-contraction

## Abstract

In this paper, we introduce the concept of $(T,g)_{F}$-contraction and investigate some fixed point theorems for such contraction in *b*-metric-like spaces. Moreover, an example is given to support one of our results, and an application is proposed.

## Introduction and preliminaries

In this paper, $\mathbb{N}$, $\mathbb{N}^{+}$ and $\mathbb{R}$ are used to denote the set of all nonnegative integer numbers, the set of all positive integer numbers, and the set of all real numbers, respectively.

The Banach contraction mapping principle in metric spaces is an important tool in nonlinear analysis, many authors have been devoting in generalizing metric spaces and the Banach contraction mapping principle. And then, many generalized metric spaces were introduced. In 2013, Alghamdi et al. [1] proposed the concept of *b*-metric-like spaces which is considered to be an interesting generalization of metric spaces, *b*-metric spaces [2] and metric-like spaces [3]. After that, some fixed point theorems were investigated by many authors [4–8]. Firstly, let us recall some definitions about *b*-metric-like spaces.

### Definition 1.1

([1])

A *b*-metric-like on a nonempty set *X* is a function $b: X\times X\rightarrow [0, +\infty)$ such that, for all $x,y,z\in X$ and a constant $s\geq1$, the following three conditions hold true:

$(b_{1})$
if $b(x,y)=0 $ then $x=y$;
$(b_{2})$
$b(x,y)=b(y,x)$;
$(b_{3})$
$b(x,z)\leq s[b(x,y)+b(y,z)]$.

The pair $(X,b)$ is then called a *b*-metric-like space with coefficient *s*.

Some concepts in *b*-metric-like spaces were introduced as follows.

Each *b*-metric-like *b* on *X* generalizes a topology $\tau_{b}$ on *X* whose base is the family of open *b*-balls $B_{b}(x,\varepsilon)=\{y\in X:|b(x,y)-b(x,x)|<\varepsilon\}$ for all $x\in X$ and $\varepsilon>0$.

A sequence $\{x_{n}\}$ in a *b*-metric-like space $(X,b)$ converges to a point $x\in X$ if and only if $b(x,x)=\lim_{n\rightarrow+\infty}b(x,x_{n})$.

A sequence $\{x_{n}\}$ in a *b*-metric-like space $(X,b)$ is called a Cauchy sequence if $\lim_{n,m\rightarrow+\infty}b(x_{m},x_{n})$ exists and is finite.

A *b*-metric-like space is called to be complete if every Cauchy sequence $\{x_{n}\}$ in *X* converges with respect to $\tau_{b}$ to a point $x\in X$ such that $\lim_{n\rightarrow+\infty}b(x,x_{n})=b(x,x)=\lim_{n,m\rightarrow+\infty}b(x_{m},x_{n})$.

Let $\mathbb{B}$ be the family of all functions *β*:$[0,\infty)\rightarrow[0,1)$ which satisfy the condition: $\lim_{n\rightarrow+\infty}\beta(t_{n})=1$ implies $\lim_{n\rightarrow+\infty}t_{n}=0$.

On the other hand, in [9], Geraghty extended the Banach contraction mapping principle in metric spaces and obtained the following theorem.

### Theorem 1.1

([9])

*Let* $(X,d)$ *be a complete metric space and* $T:X\rightarrow X$ *be a mapping*. *If* *T* *satisfies* $d(Tx,Ty)\leq \beta(d(x,y))d(x,y)$ *for any* $x,y\in X$, *where* $\beta\in \mathbb{B}$, *then* *T* *has a unique fixed point*.

Recently, many papers about generalization of Geraghty contraction appeared [10–13]. In 2017, Fulga et al. [12] introduced the concept of $\varphi_{E}$-Geraghty contraction and established a fixed point theorem for such contraction in complete metric spaces. The new Geraghty type contraction was studied on metric-like spaces in [14], and the following theorem was obtained.

### Theorem 1.2

([14])

*Let* $(X,\sigma)$ *be a complete metric*-*like space and* $T:X\rightarrow X$ *be a mapping*. *If there exists* $\beta\in \mathbb{B}$ *such that* $\sigma(Tx,Ty)\leq \beta(F(x,y))F(x,y)$ *for all* $x,y\in X$, *where* $F(x,y)=\sigma(x,y)+|\sigma(x,Tx)-\sigma(y,Ty)|$, *then* *T* *has a unique fixed point*.

In this paper, we define the new concept of $(T,g)_{F}$-contraction of Geraghty type and investigate common fixed point theorems for such contraction in b-metric-like spaces. An application about the unique solution of an integral equation is given.

## Main results

In this section, we begin with the following definitions.

### Definition 2.1

([15])

Let *X* be a nonempty set, *f* and *g* be self-mappings on *X* and $C(f,g)=\{x\in X: fx=gx\}$. The pair *f* and *g* are called to be weakly compatible if $fgx=gfx$ for all $x\in C(f,g)$. If $w=fx=gx$ for some $x\in X$, then *x* is called to be a coincidence of *f* and *g*, and *w* is called to be a point of coincidence of *f* and *g*.

### Definition 2.2

Let $(X,b)$ be a *b*-metric-like space with coefficient $s\geq 1$ and $T,g: X\rightarrow X$ be two mappings. We say that the pair $(T,g)$ is a $(T,g)_{F}$-contraction of Geraghty type if there exists $\beta\in \mathbb{B}$ such that

1 $$\begin{aligned} b(Tx,Ty)\leq \beta\bigl(F_{g}(x,y)\bigr)F_{g}(x,y) \end{aligned}$$

for all $x,y\in X$, where $F_{g}(x,y)=\frac{1}{s^{2}}[b(gx,gy)+|b(gx,Tx)-b(gy,Ty)|]$.

### Lemma 2.1

*Let* $(X,b)$ *be a b*-*metric*-*like space*, *T* *and* *g* *be self*-*mappings on* *X* *such that* $(T,g)$ *is a* $(T,g)_{F}$-*contraction of Geraghty type*. *If* $v\in X$ *is a point of coincidence of* *T* *and* *g*, *then* $b(v,v)=0$.

### Proof

Suppose that $v\in X$ is a point of coincidence of *T* and *g*, then there exists $u\in X$ such that $Tu=gu=v$. Assume $b(v,v)>0$, we get $b(v,v)=b(Tu,Tu)\leq \beta(F_{g}(u,u))F_{g}(u,u)$, since $F_{g}(u,u)=\frac{1}{s^{2}}[b(gu,gu)+|b(gu,Tu)-b(gu,Tu)|]=\frac{1}{s^{2}}b(v,v)$, then we have $b(v,v)<\frac{1}{s^{2}}b(v,v)$, which is a contradiction, hence $b(v,v)=0$. □

### Theorem 2.1

*Let* $(X,b)$ *be a* *b*-*metric*-*like space with coefficient* $s\geq 1$, $T,g:X\times X\rightarrow X$ *be two mappings with* $TX\subseteq gX$ *and* *gX* *is complete*. *If the pair* $(T,g)$ *is a* $(T,g)_{F}$-*contraction of Geraghty type*, *then* *T* *and* *g* *have a unique point of coincidence*. *In addition*, *if* *T* *and* *g* *are weakly compatible*, *then* *T* *and* *g* *have a unique common fixed point*.

### Proof

For an arbitrary $x_{0}\in X$, since $TX\subseteq gX$, we can construct a sequence $\{y_{n}\}$ by

2 $$\begin{aligned} y_{n}=gx_{n}=Tx_{n-1} \end{aligned}$$

for all $n\in \mathbb{N^{+}}$. Now, we prove that *T* and *g* have a point of coincidence. If there exists some $n_{0}\in \mathbb{N^{+}}$ such that $b(y_{n_{0}},y_{n_{0}+1})=0$, then $y_{n_{0}}=y_{n_{0}+1}$, which implies $gx_{n_{0}}=Tx_{n_{0}}$, thus, $x_{n_{0}}$ is a coincidence point of *T* and *g*, so $w_{0}=gx_{n_{0}}=Tx_{n_{0}}$ is a point of coincidence of *T* and *g*. We assume that $b(y_{n},y_{n+1})>0$ for all $n\in \mathbb{N^{+}}$. From (1), we have

3 $$\begin{aligned} b(y_{n},y_{n+1}) =&b(Tx_{n-1},Tx_{n}) \\ \leq& \beta\bigl(F_{g}(x_{n-1},x_{n}) \bigr)F_{g}(x_{n-1},x_{n}), \end{aligned}$$

where

$$\begin{aligned} F_{g}(x_{n-1},x_{n}) =&\frac{1}{s^{2}} \bigl[b(gx_{n-1},gx_{n})+ \bigl\vert b(gx_{n-1},Tx_{n-1})-b(gx_{n},Tx_{n}) \bigr\vert \bigr] \\ =&\frac{1}{s^{2}}\bigl[b(y_{n-1},y_{n})+ \bigl\vert b(y_{n-1},y_{n})-b(y_{n},y_{n+1}) \bigr\vert \bigr]. \end{aligned}$$

Assume that there exists $n_{0}\in \mathbb{N^{+}}$ such that $b(y_{n_{0}-1},y_{n_{0}})\leq b(y_{n_{0}},y_{n_{0}+1})$. By (3), we get

$$\begin{aligned} b(y_{n_{0}},y_{n_{0}+1}) =&b(Tx_{n_{0}-1},Tx_{n_{0}}) \\ \leq&\beta\bigl(F_{g}(x_{n_{0}-1},x_{n_{0}}) \bigr)F_{g}(x_{n_{0}-1},x_{n_{0}}) \\ < &F_{g}(x_{n_{0}-1},x_{n_{0}}) \\ =&\frac{1}{s^{2}}\bigl[b(gx_{n_{0}-1},gx_{n_{0}})+ \bigl\vert b(gx_{n_{0}-1},Tx_{n_{0}-1})-b(gx_{n_{0}},Tx_{n_{0}}) \bigr\vert \bigr] \\ =&\frac{1}{s^{2}}\bigl[b(y_{n_{0}-1},y_{n_{0}})+ \bigl\vert b(y_{n_{0}-1},y_{n_{0}})-b(y_{n_{0}},y_{n_{0}+1}) \bigr\vert \bigr] \\ =&\frac{1}{s^{2}}b(y_{n_{0}},y_{n_{0}+1})\leq b(y_{n_{0}},y_{n_{0}+1}), \end{aligned}$$

which is a contradiction. Thus, we obtain

4 $$\begin{aligned} b(y_{n-1},y_{n})>b(y_{n},y_{n+1}) \end{aligned}$$

for all $n\in \mathbb{N^{+}}$. Therefore, there exists $a\geq 0$ such that

5 $$\begin{aligned} \lim_{n\rightarrow+\infty}b(y_{n-1},y_{n})=a. \end{aligned}$$

(3) and (4) yield that

6 $$\begin{aligned} b(y_{n},y_{n+1}) =&b(Tx_{n-1},Tx_{n}) \\ \leq&\beta\bigl(F_{g}(x_{n-1},x_{n})\bigr)F_{g}(x_{n-1},x_{n}) \\ =&\beta\biggl[\frac{1}{s^{2}}\bigl(2b(y_{n-1},y_{n})-b(y_{n},y_{n+1}) \bigr)\biggr]\cdot\frac{1}{s^{2}}\bigl(2b(y_{n-1},y_{n})-b(y_{n},y_{n+1}) \bigr) \\ \leq&\beta\biggl[\frac{1}{s^{2}}\bigl(2b(y_{n-1},y_{n})-b(y_{n},y_{n+1}) \bigr)\biggr]\cdot\bigl(2b(y_{n-1},y_{n})-b(y_{n},y_{n+1}) \bigr) \\ < & 2b(y_{n-1},y_{n})-b(y_{n},y_{n+1}). \end{aligned}$$

Taking $n\rightarrow+\infty$ in (6), we get $\lim_{n\rightarrow+\infty}\beta[\frac{2b(y_{n-1},y_{n})-b(y_{n},y_{n+1})}{s^{2}}]=1$, hence $\lim_{n\rightarrow+\infty}\frac{2b(y_{n-1},y_{n})-b(y_{n},y_{n+1})}{s^{2}}=0$. On the other hand, $\lim_{n\rightarrow+\infty}\frac{2b(y_{n-1},y_{n})-b(y_{n},y_{n+1})}{s^{2}}=\frac{a}{s^{2}}$, therefore $a=0$. Hence,

7 $$\begin{aligned} \lim_{n\rightarrow +\infty}b(y_{n-1},y_{n})=0. \end{aligned}$$

Now, we prove

8 $$\begin{aligned} \lim_{m,n\rightarrow+\infty}b(y_{m},y_{n})=0. \end{aligned}$$

If (8) does not hold, then there exists $\varepsilon>0$, for which we can find two subsequences $\{y_{m(k)}\}$ and $\{y_{n(k)}\}$ of $\{y_{n}\}$, where $m(k)$ is the smallest index for which $m(k)>n(k)>k$ with

9 $$\begin{aligned} b(y_{m(k)},y_{n(k)})\geq \varepsilon,\qquad b(y_{m(k)-1},y_{n(k)})< \varepsilon. \end{aligned}$$

Applying (1) and (9), we have

10 $$\begin{aligned} \varepsilon \leq& b(y_{m(k)},y_{n(k)}) \\ =& b(Tx_{m(k)-1},Tx_{n(k)-1}) \\ \leq& \beta\bigl(F_{g}(x_{m(k)-1},x_{n(k)-1}) \bigr)F_{g}(x_{m(k)-1},x_{n(k)-1}) \\ < &F_{g}(x_{m(k)-1},x_{n(k)-1}), \end{aligned}$$

where

11 $$\begin{aligned} &F_{g}(x_{m(k)-1},x_{n(k)-1}) \\ &\quad =\frac{1}{s^{2}}\bigl[b(gx_{m(k)-1},gx_{n(k)-1})+ \bigl\vert b(gx_{m(k)-1},Tx_{m(k)-1})-b(gx_{n(k)-1},Tx_{n(k)-1}) \bigr\vert \bigr] \\ &\quad =\frac{1}{s^{2}}\bigl[b(y_{m(k)-1},y_{n(k)-1})+ \bigl\vert b(y_{m(k)-1},y_{m(k)})-b(y_{n(k)-1},y_{n(k)}) \bigr\vert \bigr]. \end{aligned}$$

Next, we discuss two cases.

Case I: Case of $s>1$. Applying (7), (10), and (11), we obtain

12 $$\begin{aligned} \varepsilon\leq \liminf_{n\rightarrow+\infty} \frac{1}{s^{2}}b(y_{m(k)-1},y_{n(k)-1}). \end{aligned}$$

Moreover, from (9), we have

$$\begin{aligned} b(y_{m(k)-1},y_{n(k)-1})\leq sb(y_{m(k)-1},y_{n(k)})+sb(y_{n(k)},y_{n(k)-1})< s \varepsilon+sb(y_{n(k)},y_{n(k)-1}). \end{aligned}$$

Taking $n\rightarrow +\infty$ in the above inequalities, we have

13 $$\begin{aligned} \liminf_{n\rightarrow+\infty}b(y_{m(k)-1},y_{n(k)-1}) \leq s\varepsilon. \end{aligned}$$

(12) and (13) imply $\varepsilon\leq \frac{\varepsilon}{s}$, which is a contradiction.

Case II: Case of $s=1$. From (9), we have

14 $$\begin{aligned} \varepsilon \leq &b(y_{m(k)},y_{n(k)}) \\ \leq& b(y_{m(k)},y_{m(k)-1})+ b(y_{m(k)-1},y_{n(k)-1})+b(y_{n(k)-1},y_{n(k)}) \\ \leq& b(y_{m(k)},y_{m(k)-1})+ b(y_{m(k)-1},y_{n(k)})+2b(y_{n(k)-1},y_{n(k)}) \\ < &b(y_{m(k)},y_{m(k)-1})+\varepsilon+2b(y_{n(k)-1},y_{n(k)}). \end{aligned}$$

By (7), taking $n\rightarrow +\infty$ in (14), we have

15 $$\begin{aligned} \lim_{n\rightarrow+\infty}b(y_{m(k)-1},y_{n(k)-1})= \varepsilon. \end{aligned}$$

Since $s=1$, by (10) and (11), we have

16 $$\begin{aligned} \varepsilon \leq &\beta\bigl(F_{g}(x_{m(k)-1},x_{n(k)-1}) \bigr)F_{g}(x_{m(k)-1},x_{n(k)-1}) \\ < &b(y_{m(k)-1},y_{n(k)-1})+ \bigl\vert b(y_{m(k)-1},y_{m(k)})-b(y_{n(k)-1},y_{n(k)}) \bigr\vert . \end{aligned}$$

(7), (15) and (16) yield

17 $$\begin{aligned} \lim_{n\rightarrow+\infty}\beta\bigl(F_{g}(x_{m(k)-1},x_{n(k)-1}) \bigr)F_{g}(x_{m(k)-1},x_{n(k)-1})= \varepsilon. \end{aligned}$$

From (11) and (15), and taking $s=1$ into account, we get $\lim_{n\rightarrow+\infty}F_{g}(x_{m(k)-1},x_{n(k)-1})= \varepsilon$, which together with (17) implies

$$\begin{aligned} \lim_{n\rightarrow+\infty}\beta\bigl(F_{g}(x_{m(k)-1},x_{n(k)-1}) \bigr)=1, \end{aligned}$$

thus $\lim_{n\rightarrow+\infty}F_{g}(x_{m(k)-1},x_{n(k)-1})=0$, which is contradictive with $\lim_{n\rightarrow+\infty}F_{g}(x_{m(k)-1}, x_{n(k)-1})= \varepsilon$.

From the above discussions, we get that (8) holds. Therefore, the sequence $\{y_{n}\}=\{gx_{n}\}$ is a Cauchy sequence in *gX*. Since *gX* is complete, then there exist $v,u\in X$ such that $v=gu$, and the following equalities hold:

18 $$\begin{aligned} \lim_{n,m\rightarrow+\infty}b(y_{n},v)=b(v,v)=\lim _{n,m\rightarrow+\infty}b(y_{n},y_{m})=\lim _{n\rightarrow+\infty}b(y_{n},gu)=0. \end{aligned}$$

By (1), we have

19 $$\begin{aligned} b(y_{n},Tu)=b(Tx_{n-1},Tu)\leq\beta \bigl(F_{g}(x_{n-1},u)\bigr)F_{g}(x_{n-1},u)< F_{g}(x_{n-1},u), \end{aligned}$$

where

20 $$\begin{aligned} F_{g}(x_{n-1},u) =&\frac{1}{s^{2}} \bigl[b(gx_{n-1},gu)+ \bigl\vert b(gx_{n-1},Tx_{n-1})-b(gu,Tu) \bigr\vert \bigr] \\ =&\frac{1}{s^{2}}\bigl[b(y_{n-1},v)+ \bigl\vert b(y_{n-1},y_{n})-b(v,Tu) \bigr\vert \bigr]. \end{aligned}$$

Next, we prove $b(Tu,v)=0$ in two cases:

Case I. $s>1$. Suppose $b(Tu,v)>0$. Letting $n\rightarrow +\infty$ in (19), applying (20), we obtain

21 $$\begin{aligned} \liminf_{n\rightarrow+\infty}b(y_{n},Tu)\leq \frac{1}{s^{2}}b(v,Tu). \end{aligned}$$

By the triangle inequality, we get $b(v,Tu)\leq sb(y_{n},v)+sb(y_{n},Tu)$, which yields

22 $$\begin{aligned} b(v,Tu)\leq s\liminf_{n\rightarrow+\infty}b(y_{n},Tu). \end{aligned}$$

Applying (22), we have $\liminf_{n\rightarrow\infty}b(y_{n},Tu)\geq \frac{1}{s}b(v,Tu)>0$. From (21) and (22), we get $b(v,Tu)\leq\frac{1}{s}b(v,Tu)< b(v,Tu)$, this is a contradiction, therefore $b(Tu,v)=0$.

Case II. $s=1$. Taking $n\rightarrow +\infty$ in (20), and taking $s=1$ into account, we obtain

23 $$\begin{aligned} \lim_{n\rightarrow+\infty}F_{g}(x_{n-1},u)=b(v,Tu). \end{aligned}$$

On the other hand, from (1), we have

24 $$\begin{aligned} b(v,Tu) \leq& b(v,y_{n})+b(y_{n},Tu) \\ =& b(v,y_{n})+b(Tx_{n-1},Tu) \\ \leq& b(v,y_{n})+\beta\bigl(F_{g}(x_{n-1},u) \bigr)F_{g}(x_{n-1},u) \\ < & b(v,y_{n})+F_{g}(x_{n-1},u). \end{aligned}$$

Letting $n\rightarrow +\infty$ in (24), by (23), we get $\lim_{n\rightarrow+\infty}\beta(F_{g}(x_{n-1},u))=1$, hence $\lim_{n\rightarrow+\infty}F_{g}(x_{n-1},u)=0$, by (23), we get $b(Tu,v)=0$. The above two cases mean $b(Tu,v)=0$, which implies $Tu=v$, thus $Tu=v=gu$. Therefore, *T* and *g* have a coincidence point *u*, and *v* is a point of coincidence of *T* and *g*. By Lemma 2.1, we get $b(v,v)=0$. Suppose that $v_{1}$ is also a point of coincidence of *T* and *g*, then we can find $u_{1}\in X$ such that $Tu_{1}=v_{1}=gu_{1}$ and $b(v_{1},v_{1})=0$. Now, we prove $b(v,v_{1})=0$ by contradiction. Suppose $b(v,v_{1})>0$, applying (1), we have

25 $$\begin{aligned} b(v,v_{1}) =& b(Tu,Tu_{1}) \leq \beta\bigl(F_{g}(u,u_{1})\bigr)F_{g}(u,u_{1}) < F_{g}(u,u_{1}), \end{aligned}$$

where

26 $$\begin{aligned} F_{g}(u,u_{1}) =&\frac{1}{s^{2}} \bigl[b(gu,gu_{1})+ \bigl\vert b(gu,Tu)-b(gu_{1},Tu_{1}) \bigr\vert \bigr] \\ =&\frac{1}{s^{2}}\bigl[b(v,v_{1})+ \bigl\vert b(v,v)-b(v_{1},v_{1}) \bigr\vert \bigr] \\ =&\frac{1}{s^{2}}b(v,v_{1}). \end{aligned}$$

From (25) and (26), we obtain $b(v,v_{1})<\frac{1}{s^{2}}b(v,v_{1})$, which is a contradiction, thus $b(v,v_{1})=0$, which implies $v=v_{1}$, therefore *T* and *g* have a unique point of coincidence. Moreover, *T* and *g* are weakly compatible, then we have $Tv=gv$. Let $Tv=gv=\omega$. From the uniqueness of the point of coincidence, we have $Tv=gv=\omega=v$, that is, $Tv=gv=v$. Therefore, *T* and *g* have a unique common fixed point. □

Letting $g=I_{x}$ (identity mapping) in Theorem 2.1, we can get the following corollary.

### Corollary 2.1

*Let* $(X,b)$ *be a complete b*-*metric*-*like space with coefficient* $s\geq1$, *and* $T:X\rightarrow X$ *be a mapping*. *If there exists* $\beta\in \mathbb{B}$ *such that* $b(Tx,Ty)\leq \beta(F(x,y))F(x,y)$ *for any* $x,y\in X$, *where* $F(x,y)=\frac{1}{s^{2}}[b(x,y)+|b(x,Tx)-b(y,Ty)|]$, *then* *T* *has a unique fixed point*.

Taking $s=1$ in Corollary 2.1, we have the following corollary.

### Corollary 2.2

*Let* $(X,b)$ *be a complete metric*-*like space and* $T:X\rightarrow X$ *be a mapping*. *If there exists* $\beta\in \mathbb{B}$ *such that* $b(Tx,Ty)\leq \beta(F(x,y))F(x,y)$ *for any* $x,y\in X$, *where* $F(x,y)=b(x,y)+|b(x,Tx)-b(y,Ty)|$, *then* *T* *has a unique fixed point*.

### Remark 2.1

Corollary 2.2 is Theorem 1.2, which implies that Theorem 2.1 is the generalization of Theorem 1.2.

Taking $s=1$ in Theorem 2.1, we have the following corollary.

### Corollary 2.3

*Let* $(X,b)$ *be a metric*-*like space and* $T,g:X\times X\rightarrow X$ *be two mappings with* $TX\subseteq gX$ *and* *gX* *is complete*. *Suppose that there exists* $\beta\in \mathbb{B}$ *such that*

$$\begin{aligned} b(Tx,Ty)\leq \beta\bigl(F_{g}(x,y)\bigr)F_{g}(x,y), \end{aligned}$$

*where* $F_{g}(x,y)=b(gx,gy)+|b(gx,Tx)-b(gy,Ty)|$. *Then* *T* *and* *g* *have a unique point of coincidence*. *In addition*, *if* *T* *and* *g* *are weakly compatible*, *then* *T* *and* *g* *have a unique common fixed point*.

Now, we use an example to illustrate the validity of our main result.

### Example 2.1

Let $X=\{0,1,2\}$. Define $b: X \times X\rightarrow \mathbb{R}$ by $b(0,0)=0$, $b(1,1)=3$, $b(2,2)=1$, $b(0,1)=b(1,0)=8$, $b(0,2)=b(2,0)=1$, $b(1,2)=b(2,1)=4$. It is easy to prove that $(X,b)$ is a complete b-metric-like space with coefficient $s=\frac{8}{5}$. Consider $T: X\rightarrow X$ as $T0=0, T1=2, T2=0$. Take

$$\begin{aligned} \beta(t)=\textstyle\begin{cases}\frac{1}{1+\frac{1}{100}t}, & t>0, \\\frac{1}{3}, &t=0.\end{cases}\displaystyle \end{aligned}$$

By the following cases, we prove $b(Tx,Ty)\leq \beta(F(x,y))F(x,y)$ for any $x,y\in X$, where $F(x,y)=\frac{1}{s^{2}}[b(x,y)+|b(x,Tx)-b(y,Ty)|]$.

Case 1: $(x,y)=(0,0)$, $(x,y)=(2,2)$, $(x,y)=(0,2)$. Since $b(T0,T0)=b(0,0)=0$, $b(T2,T2)=b(0,0)=0$, $b(T0,T2)= b(0,0)=0$, then $b(Tx,Ty)\leq \beta(F(x,y))F(x,y)$ holds for $(x,y)=(0,0)$, $(x,y)=(2,2)$, $(x,y)=(0,2)$.

Case 2: $(x,y)=(0,1)$.

We get $b(T0,T1)=b(0,2)=1$ and $F(0,1)=\frac{25}{64}[b(0,1)+|b(0,T0)-b(1,T1)|]=\frac{300}{64}$. Hence $b(T0,T1)=1<\beta (F(0,1))F(0,1)=\frac{1}{1+\frac{1}{100}\frac{300}{64}}\frac{300}{64}=\frac{300}{94}$.

Case 3: $(x,y)=(1,1)$.

We get $b(T1,T1)=b(2,2)=1$ and $F(1,1)=\frac{25}{64}[b(1,1)+|b(1,T1)-b(1,T1)|]=\frac{75}{64}$. Hence $b(T1,T1)=1<\beta (F(1,1))F(1,1)=\frac{1}{1+\frac{1}{100}\frac{75}{64}}\frac{75}{64}=\frac{750}{715}$.

Case 4: $(x,y)=(1,2)$.

We get $b(T1,T2)=b(2,0)=1$ and $F(1,2)=\frac{25}{64}[b(1,2)+|b(1,T1)-b(2,T2)|]=\frac{175}{64}$. Hence $b(T1,T2)=1<\beta (F(0,1))F(0,1)=\frac{1}{1+\frac{1}{100}\frac{175}{64}}\frac{175}{64}=\frac{1750}{815}$.

From the above discussions, we know that $b(Tx,Ty)\leq \beta(F(x,y))F(x,y)$ for any $x,y\in X$, where $F(x,y)=\frac{1}{s^{2}}[b(x,y)+|b(x,Tx)-b(y,Ty)|]$. By Corollary 2.1, we obtain that *T* has a unique fixed point, 0 is the unique fixed point of *T*.

## Existence of a solution for an integral equation

Consider the following integral equation:

27 $$\begin{aligned} x(t)= \int_{0}^{1}K\bigl(t,r,x(r)\bigr)\,dr, \end{aligned}$$

where $K:[0,1]\times [0,1]\times R\rightarrow R$. The purpose of this section is to present an existence theorem of the solution about (27). Let $X=C[0,1]$ be the set of continuous real functions defined on $[0,1]$. We endow *X* with the b-metric-like:

$$\begin{aligned} b(u,v)= \Vert u-v \Vert _{\infty}=\max_{t\in[0,1]}\bigl( \bigl\vert u(t) \bigr\vert + \bigl\vert v(t) \bigr\vert \bigr)^{p}\quad \mbox{for all }u,v\in X, \end{aligned}$$

where $p\geq1$. Obviously, $(X,b)$ is a complete b-metric-like space with coefficient $s=2^{p-1}$.

Let $f(x)(t)=\int_{0}^{1}K(t,r,x(r))\,dr$ for all $x\in X$ and for all $t\in [0,1]$. Then the existence of a solution to (27) is equivalent to the existence of a fixed point of *f*.

Now, we prove the following result.

### Theorem 3.1

*Suppose that the following hypotheses hold*:

(i) $K:[0,1]\times [0,1]\times R\rightarrow R$ *is continuous*;
(ii) *For all* $t,r\in [0,1]$, *there exists continuous* $\xi:[0,1]\times [0,1]\rightarrow [0,+\infty)$ *such that*
  28 $$\begin{aligned} \bigl\vert K\bigl(t,r,x(r)\bigr) \bigr\vert \leq\xi(t,r) \bigl\vert x(r) \bigr\vert . \end{aligned}$$
(iii) *There exists* $\beta\in \mathbb{B}$ *such that*
  29 $$\begin{aligned} &\sup_{t\in [0,1]} \int_{0}^{1}\xi(t,r)\,dr \\ &\quad \leq \sqrt[p]{\frac{1}{2^{2p-2}}\beta\biggl[\frac{1}{2^{2p-2}}\bigl( \Vert x-y \Vert _{\infty}+ \bigl\vert \Vert x-fx \Vert _{\infty}- \Vert y-fy \Vert _{\infty}\bigr\vert \bigr) \biggr]}. \end{aligned}$$

*Then the integral equation* (27) *has a unique solution* $x\in X$.

### Proof

From (28) and (29), for all $t\in [0,1]$, we have

$$\begin{aligned} & \bigl( \bigl\vert f(x) (t) \bigr\vert + \bigl\vert f(y) (t) \bigr\vert \bigr)^{p} \\ &\quad = \biggl( \biggl\vert \int_{0}^{1}K\bigl(t,r,x(r)\bigr)\,dr \biggr\vert + \biggl\vert \int_{0}^{1}K\bigl(t,r,y(r)\bigr)\,dr \biggr\vert \biggr)^{p} \\ &\quad \leq \biggl( \int_{0}^{1} \bigl\vert K\bigl(t,r,x(r)\bigr) \bigr\vert \,dr+ \int_{0}^{1} \bigl\vert K\bigl(t,r,y(r)\bigr) \bigr\vert \,dr \biggr)^{p} \\ &\quad = \biggl( \int_{0}^{1}\bigl( \bigl\vert K\bigl(t,r,x(r) \bigr) \bigr\vert + \bigl\vert K\bigl(t,r,y(r)\bigr) \bigr\vert \bigr)\,dr \biggr)^{p} \\ &\quad \leq \biggl( \int_{0}^{1}\xi(t,r) \bigl( \bigl\vert x(r) \bigr\vert + \bigl\vert y(r) \bigr\vert \bigr)\,dr \biggr)^{p} \\ &\quad = \biggl( \int_{0}^{1}\xi(t,r) \bigl(\bigl( \bigl\vert x(r) \bigr\vert + \bigl\vert y(r) \bigr\vert \bigr)^{p} \bigr)^{\frac{1}{p}}\biggr)\,dr )^{p} \\ &\quad \leq \biggl( \int_{0}^{1}{\xi(t,r)\bigl[b\bigl(x(\cdot),y(\cdot) \bigr)\bigr]^{\frac{1}{p}}\biggr)\,dr} )^{p} \\ &\quad = \Vert x-y \Vert _{\infty}\biggl( \int_{0}^{1}\xi(t,r)\,dr \biggr)^{p} \\ &\quad \leq \Vert x-y \Vert _{\infty}\frac{1}{2^{2p-2}}\beta\biggl[ \frac{1}{2^{2p-2}}\bigl( \Vert x-y \Vert _{\infty}+ \bigl\vert \Vert x-fx \Vert _{\infty}- \Vert y-fy \Vert _{\infty}\bigr\vert \bigr)\biggr] \\ &\quad \leq F(x,y)\beta\bigl(F(x,y)\bigr), \end{aligned}$$

where $F(x,y)=\frac{1}{2^{2p-2}}(\|x-y\|_{\infty}+|\|x-fx\|_{\infty}-\|y-fy\|_{\infty}|)$. Then we have $\|f(x)-f(y)\|_{\infty}\leq F(x,y)\beta(F(x,y))$. Therefore, we get $b(f(x),f(y))\leq F(x,y)\beta(F(x,y))$ for all $x,y\in X$.

From the above, we can see that all the conditions of Corollary 2.1 hold and *f* has a unique fixed point $x\in X$, which means that *x* is the unique solution for the integral equation (27). □

## Conclusion

In this paper, we introduce a new concept of $(T,g)_{F}$-contraction in b-metric-like spaces and investigate some fixed point theorems about such contraction. Our results generalize Theorem 2.1 in [14]. At the same time, an application about our theorem is also proposed.
